# Supplementary material for: Environmental factors of food insecurity in adolescents: A scoping review protocol
Source: PLoS One. 2023 Nov 21;18(11):e0294506. doi: 10.1371/journal.pone.0294506 (PMC10662750; doi:10.1371/journal.pone.0294506)
Supplement: S1 Table — (DOCX) [file pone.0294506.s002.docx]

| **Database** | **Search strategy** | |
| --- | --- | --- |
| Pubmed/Medline | **#** | **Search statement** |
|  | 1 | ((food security[MeSH Terms]) OR (food insecurity[MeSH Terms]) OR (food supply[MeSH Terms]) OR (food access[Title/Abstract]) OR (food poverty[Title/Abstract])) |
|  | 2 | ((environmental factors[Title/Abstract]) OR (sociological factors[MeSH Terms]) OR (government[MeSH Terms])) |
|  | 3 | ((adolescent[MeSH Terms]) OR (child[MeSH Terms])) |
|  | 4 | 1 AND 2 AND 3 |
|  | 5 | Limit 4 to English, Portuguese, Spanish |
| EMBASE |  | ('food insecurity'/exp OR 'food security'/exp OR 'food access'/exp) AND ('adolescent'/mj OR 'child'/mj) AND ('social aspects and related phenomena'/exp OR 'family life'/exp OR 'policy'/exp) AND ([english]/lim OR [portuguese]/lim OR [spanish]/lim) |
| BVS | **#** | **Search statement** |
|  | 1 | ("food security" OR "food insecurity" OR "food supply") |
|  | 2 | ("adolescent" OR "child") |
|  | 3 | 1 AND 2 |
|  | 4 | Limit 3 to English, Portuguese, Spanish |
| EBSCOHost | **#** | **Search statement** |
|  | 1 | (food insecurity OR food security OR food supply OR food access) |
|  | 2 | (neighb#rhood OR community) |
|  | 3 | (adolescent* OR child*) |
|  | 4 | (nutritional status OR obesity OR overweight OR asthma OR HIV OR homeless* OR mental health) |
|  | 5 | (1 AND 2 AND 3) NOT 4 |
|  | 6 | Limit 5 to English, Portuguese, Spanish, full text, and academic publications. |
| Cochrane |  | ("food security" OR "food inscurity" OR "food access" OR "food supply") AND ("child" OR "adolescent") in Title Abstract Keyword |
| Scopus |  | TITLE-ABS-KEY (( "food security" OR "food insecurity" ) AND ( "adolescent*" OR "child*" ) AND ( "neighb#rhood" OR "community" ) ) AND NOT ( "food safety" OR "obesity" OR "overweight" OR "nutritional status" ) AND ( LIMIT-TO ( LANGUAGE , "English" ) OR LIMIT-TO ( LANGUAGE , "Spanish" ) OR LIMIT-TO ( LANGUAGE , "Portuguese" )) |
| Web of Science | **#** | **Search statement** |
|  | 1 | TS=(food insecurity OR food security) AND TS=(adolescent* OR child*) AND TS=(neighb*rhood OR community) NOT TS=(food safety OR overweight OR obesity) |
|  | 2 | Limit 1 to English, Portuguese, Spanish. Also exclude MEDLINE. |
| Grey Literature Report |  | Search will be performed using the key terms “food security” or “food insecurity” within the title to ensure that the focus of the grey literature available is on such terms. |
